# Ultrafiltration and cardiopulmonary bypass associated acute kidney injury: A systematic review and meta‐analysis

**DOI:** 10.1002/clc.23750

**Published:** 2021-11-27

**Authors:** Omneya A. Kandil, Karam R. Motawea, Edward Darling, Jeffrey B. Riley, Jaffer Shah, Mohamed Abdalla Mohamed Elashhat, Bruce Searles, Hani Aiash

**Affiliations:** ^1^ Faculty of Medicine Alexandria University Alexandria Egypt; ^2^ Department of Cardiovascular Perfusion State University of New York Upstate Medical University Syracuse New York USA; ^3^ Medical Research Center Kateb University Kabul Afghanistan; ^4^ Aswan Heart Center (Magdy Yacoub Foundation) Aswan Governorate Aswan Egypt; ^5^ Department of Family Medicine, Faculty of Medicine Suez Canal University Ismailia Egypt; ^6^ Department of Surgery State University of New York Upstate Medical University Syracuse New York USA

**Keywords:** acute kidney injury, cardiac surgery, cardiopulmonary bypass, fluid management, ultrafiltration

## Abstract

**Background:**

Cardiopulmonary bypass is known to raise the risk of acute kidney injury (AKI). Previous studies have identified numerous risk factors of cardiopulmonary bypass including the possible impact of perioperative ultrafiltration. However, the association between ultrafiltration (UF) and AKI remains conflicting. Thus, we conducted a meta‐analysis to further examine the relationship between UF and AKI.

**Hypothesis:**

Ultrafiltration during cardiac surgery increases the risk of developping Acute kidney Injury.

**Methods:**

We searched PubMed, Web of Science, EBSCO, and SCOPUS through July 2021. The RevMan (version 5.4) software was used to calculate the pooled risk ratios (RRs) and mean differences along with their associated confidence intervals (95% CI).

**Results:**

We identified 12 studies with a total of 8005 patients. There was no statistically significant difference in the incidence of AKI between the group who underwent UF and the control group who did not (RR = 0.90, 95% CI = 0.64−1). Subgroup analysis on patients with previous renal insufficiency also yielded nonsignificant difference (RR = 0.84, 95% CI = 0.53 −1.33, *p* = .47). Subgroup analysis based on volume of ultrafiltrate removed (> or <2900 ml) was not significant and did not increase the AKI risk as predicted (RR = 0.82, 95% CI = 0.63 −1.07, *p*  = .15). We also did subgroup analysis according to the type of UF and again no significant difference in AKI incidence between UF groups and controls was observed in either the conventional ultrafiltration (CUF), modified ultrafiltration (MUF), zero‐balanced ultrafiltration (ZBUF), or combined MUF and CUF subgroups.

**Conclusion:**

UF in cardiac surgery is not associated with increased AKI incidence and may be safely used even in baseline chronic injury patients.

## 1 INTRODUCTION

Cardiopulmonary bypass (CPB)‐associated AKI occurs in 18.2% of adult patients who undergo CPB and is associated with a twofold increase in early mortality.^1^ Risks for CPB‐associated AKI have been classified as patient‐related and procedure‐related factors.^2^

Procedure‐related factors include systemic inflammatory response, anemia, oxygen delivery, coagulopathy subsequent to foreign surface exposure, and hemodilution associated with the use of a priming solution leading to renal vasoconstriction, and impaired oxygenation including the typically nonpulsatile flow used in CPB.

Ultrafiltration (UF) is a technique commonly used during CPB for volume management and/or filtration of blood to reduce deleterious components.^3^ An ultrafilter can be incorporated into a CPB circuit and plasma water, and its soluble components are removed as blood passes through the ultrafilter fibers. Common UF techniques include modified ultrafiltration (MUF), conventional ultrafiltration (CUF), and zero‐balanced ultrafiltration (ZBUF). All of these techniques share a common goal: blood concentration, filtration, and the balancing of shifts in the electrolyte plasma concentration as potassium overload, thus protecting the kidney and avoiding homologous blood transfusions.^4^, ^5^

Some studies suggest that the use of UF during CPB to remove excessive fluid is not renal protective and may even lead to kidney damage if the fluid removed is more than what is needed.^3^ Furthermore, recent reviews warn that we should limit UF in patients with reduced kidney function to prevent AKI.^4^

Some studies have set a limit to the volume of ultrafiltrate removed above which AKI can occur to 2900 ml (knowing that 2200 ml is equivalent = 32 ml/kg in an average 70 kg adult).^5^ The aim of our present study is to investigate if ultrafiltration is associated with an increased risk of AKI and the safety of its use in patients with previous kidney problems. The present study will also examine if the removal of an ultrafiltrate volume above the 2900 ml suggested limit is associated with an increased risk of AKI.

## 2 METHODS

### 2.1 Search and identification of studies

A comprehensive literature search was carried out on the following databases: PUBMED, WOS, EBSCO, and SCOPUS in July 2021. Search terms used were (hemofiltration OR ultrafiltration OR MUF OR N‐MUF OR fluid management OR CUF) AND (CBP OR CABG OR cardiac surgery) AND (AKI OR ARF OR kidney failure OR clinical outcomes).

### 2.2 Selection process and inclusion criteria

Yielded results from databases were imported into Covidence.^5^

From the searches, we reviewed the title and abstract of each paper and retrieved potentially relevant references. Following this initial screening, we obtained the full text of potentially relevant studies and did the full‐text screening for the papers using predetermined inclusion criteria, which are any trial or observational study on patients who underwent any type of filtration procedure during cardiac surgery. We excluded case reports and non‐English articles.

### 2.3 Data extraction

Details about the occurrence of AKI in the study groups along with the volume of filtrate removed and type of ultrafiltration used were extracted for subsequent analysis. In addition to study design, participant characteristics and study setting were also extracted to be presented in tables.

### 2.4 Statistical analysis

A meta‐analysis was carried out comparing the occurrence of AKI in patients who underwent cardiac surgery and received ultrafiltration and controls who did not receive ultrafiltration.

RevMan 5.4 was used to calculate the pooled risk ratios (RRs) along with their confidence intervals (CI). We used random‐effects model when we observed significant heterogeneity, and when heterogeneity was not solved by random effects, we did the leave one out test. Our analysis was reviewed following the PRISMA Statement checklist to ensure its high quality.

## 3 RESULTS

### 3.1 Study inclusion

**Figure 1:**
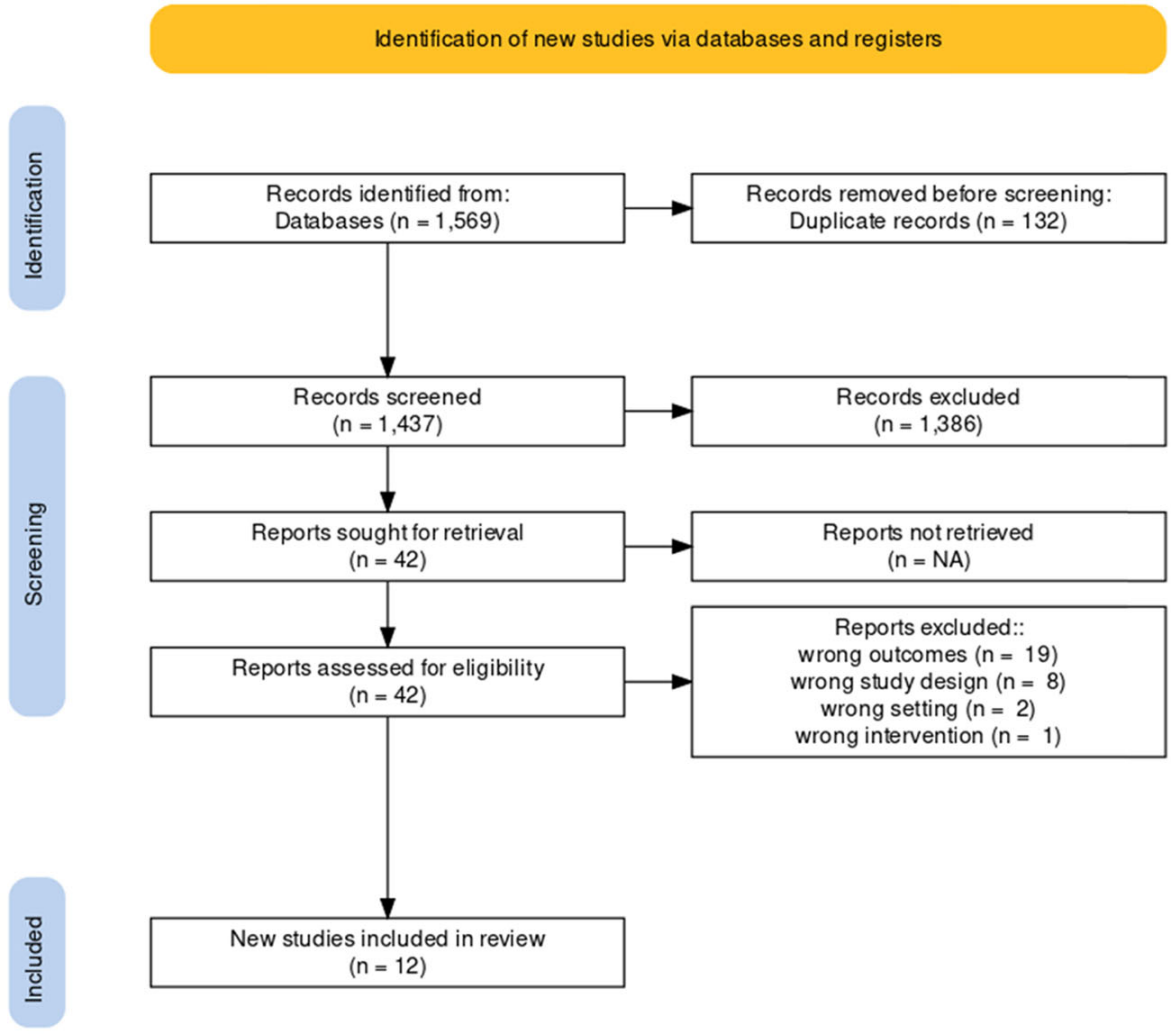
PRISMA flow chart

After a complete search of the literature, 1569 publications resulted and became 1437 after the removal of duplicates. Of these, 51 were eligible for full‐text screening after performing title and abstract screening and excluding 1386 papers that were irrelevant to our investigation. After the full‐text screening, 12 studies were eligible for inclusion in the meta‐analysis, as shown in the Prisma flow chart in (Figure 1). The included 12 studies contained data about the incidence of acute kidney injury (AKI) in patients undergoing ultrafiltration and control patients with no ultrafiltration. The summary of the included studies is shown in Table 1.

**Table 1:** Summary of the included studies

ID	Study design	Type of filtration	Participants	Volume of filtrate (mean)	Study highlights/conclusions
Luciani et al. (2001)^6^	Prospective randomized	MUF	284 in MUF versus 289 in control	18 ml/kg (1.3 L/patient)	Most but not all morbid events listed in the miscellaneous group were less common among patients having modified ultrafiltration, including postoperative hemorrhage requiring surgical reexploration, acute renal failure needing dialysis, and gastrointestinal complications. Only the difference in the latter, however, reached statistical significance
Raman et al. (2003)^7^	Retrospective study	CUF	61 patients with hemofiltration during CPB versus 57 patients without hemofiltration	3400 ml/patient	Renal dysfunction 6 (9.8%) in hemofilter group versus 10 (17.5%) in nonhemofilter group
Papadopoulos et al. (2013)^8^	Prospective, randomized trial	N‐MUF	25 patients underwent N MUF versus 25 patients did not	3000 ml/patient	No significant differences between the two groups were observed in terms of the incidence of surgical reexploration for bleeding (*n* = 2 in each group, *p* = 1.0), the incidence of wound infection (N‐MUF: 5, control‐group: 4, *p* = .15) and the incidence of acute renal failure (creatinine level >200 μg/l) (*n* = 2 in each group, *p* = 1.0)
Zhang et al. (2009)^9^	Randomized trial	SBUF	60 patients with SBUF versus 60 patients in control	Not mentioned	Subzero‐balanced ultrafiltration during cardiopulmonary bypass can effectively decrease the patients' hospital morbidity and the volume of blood transfusion: it also may promote early postoperative recovery of patients
Kuntz et al. (2006)^10^	Prospective randomized trial	CUF	49 patients in CUF group versus 47 in control	5.5 L/patient)	No significant differences in pre‐ or postoperative creatinine values were observed. Aggressive CUF can be safely used during cardiopulmonary bypass in the adult population to reduce fluid accumulation and elevate bypass hematocrit without affecting bypass or intraoperative urine production
Babka et al. (1997)^11^	Prospective study	CUF	30 patients with ultrafiltration versus 30 patients without	2510 ml/patient	The postoperative profiles of these patients revealed no new myocardial infarctions, stroke, or renal insufficiency in either group
Foroughi et al. (2014)^12^	Prospective randomized	Combined	87 in hemofilter versus 72 in no hemofilter	3532.65 ml	Routine use of ultrafiltration during cardiac surgery offers no advantages in renal protection and reduction of AKI incidence
Musleh et al. (2009)^13^	Prospective randomized	CUF	40 patients in hemofiltration versus 39 patients without hemofiltration	15 ml/kg	Use of hemofiltration during CPB was found not to be protective against renal dysfunction (*p* < .071)
Matata et al. (2015)^14^	Randomized controlled trial	ZBUF	97 patients in ZBUF versus 102 in control	8625 ml/patient	Z‐BUF during bypass surgery is associated with significant reductions in morbidity and biomarkers of CPB‐induced acute kidney injury soon after CPB, which are indicative of clearance of inflammatory/immune mediator from the circulation
Paugh et al. (2015)^15^	Retrospective	CUF	1364 in CUF group versus 5045 in non CUF	1365 ml/patient	Patients exposed to CUF had a higher adjusted risk of AKI. Clinical teams should consider lower volumes of CUF among patients with low creatinine clearance to minimize the risk of AKI
Roscitano et al. (2009)^16^	Retrospective	CUF	40 underwent CABG with CPB and CVVH versus 44 who had on‐pump CABG without CVVH	Not mentioned	We used CVVH during CPB and found that these patients had better postoperative renal function than those undergoing CABG on CPB without hemofiltration. As reported by others, OPCAB was not related to a deterioration of renal function, but our results showed an advantage of intraoperative CVVH over OPCAB, in terms of renal function
El‐Tahan et al. (2010)^17^	A prospective, randomized double‐blinded placebo study	Combined	30 patients underwent CUF versus 30 underwent CUF and MUF	3449.8 ml/patient	There were no differences between groups in the frequency of perioperative bleeding (either from the surgical site or hematemesis), coagulopathy, pulmonary, renal, new‐onset or worsening of ascites, encephalopathy, infection, or wound complications

Abbreviations: AKI, acute kidney injury; CPB, cardiopulmonary bypass; CUF, conventional ultrafiltration; MUF, modified ultrafiltration.

**Figure 2:**
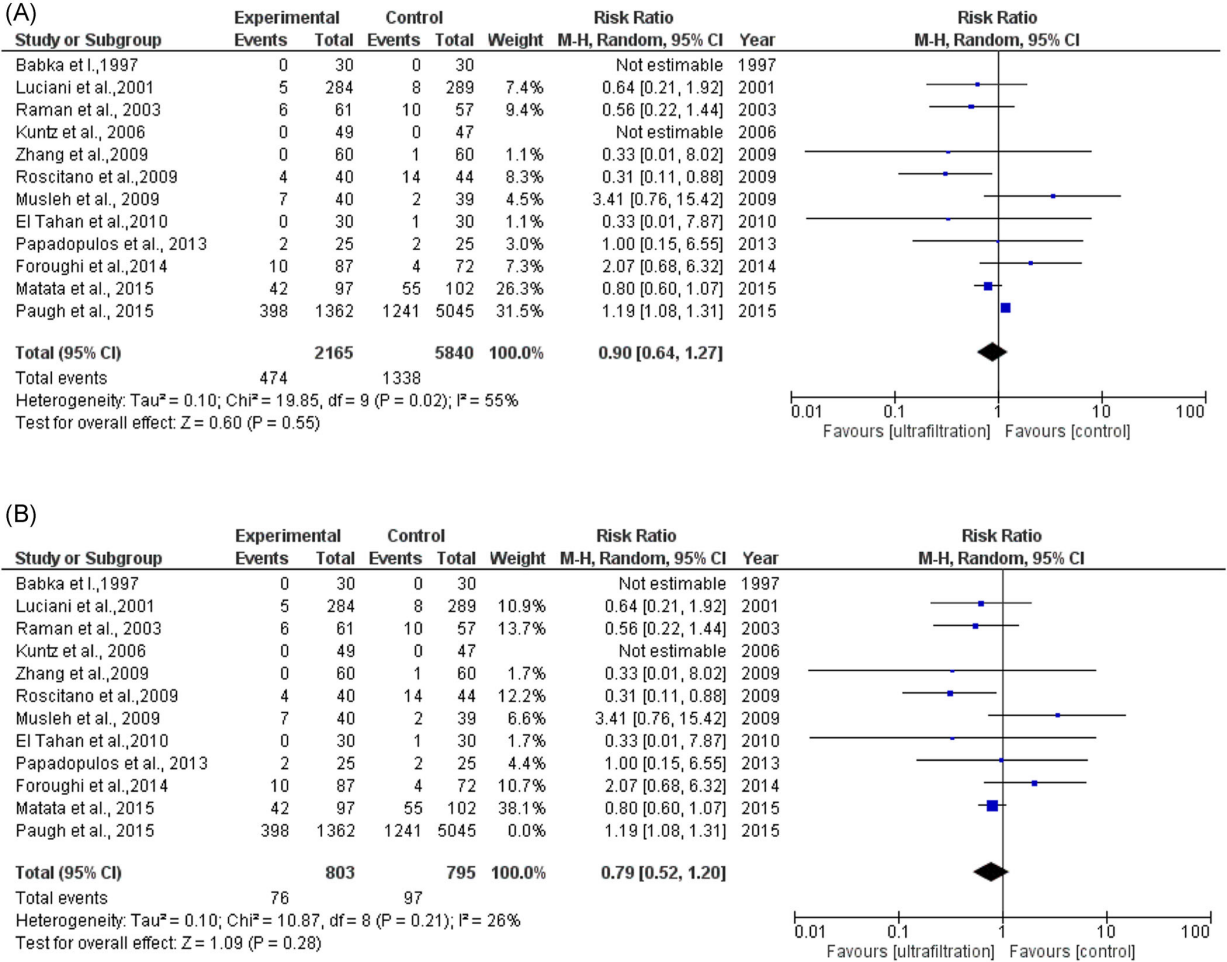
Forest plot of UF and AKI for all studies and with one study excluded. (A) Forest plot of UF and AKI for all studies. (B) Forest plot of UF and AKI with one study omitted. AKI, acute kidney injury; UF, ultrafiltration

**Figure 3:**
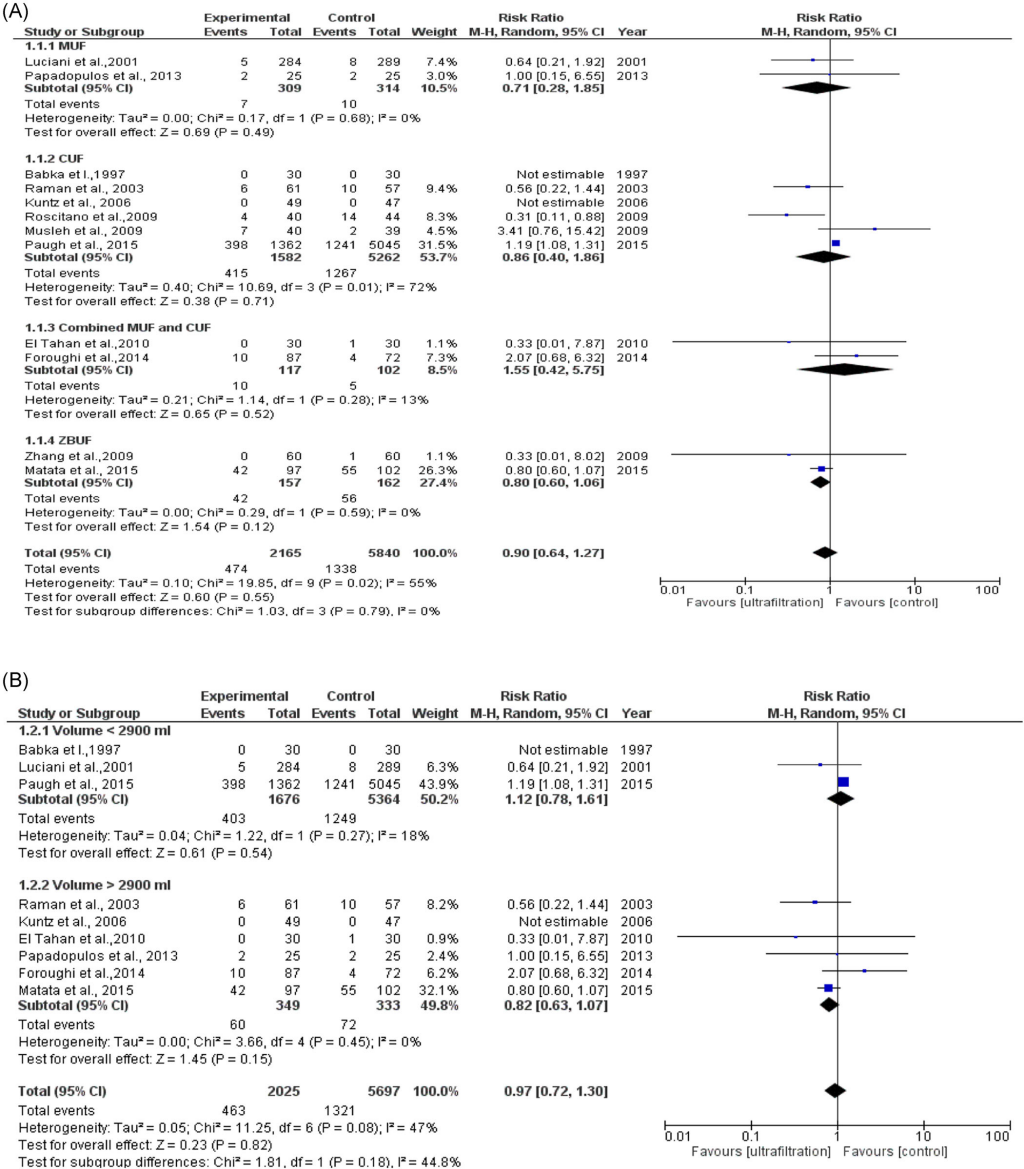
Forest plot of UF and AKI subgroup analysis according to technique and quantity of volume removed. (A) UF and AKI according to technique (B) UF and AKI according to volume removed. AKI, acute kidney injury; UF, ultrafiltration

**Figure 4:**
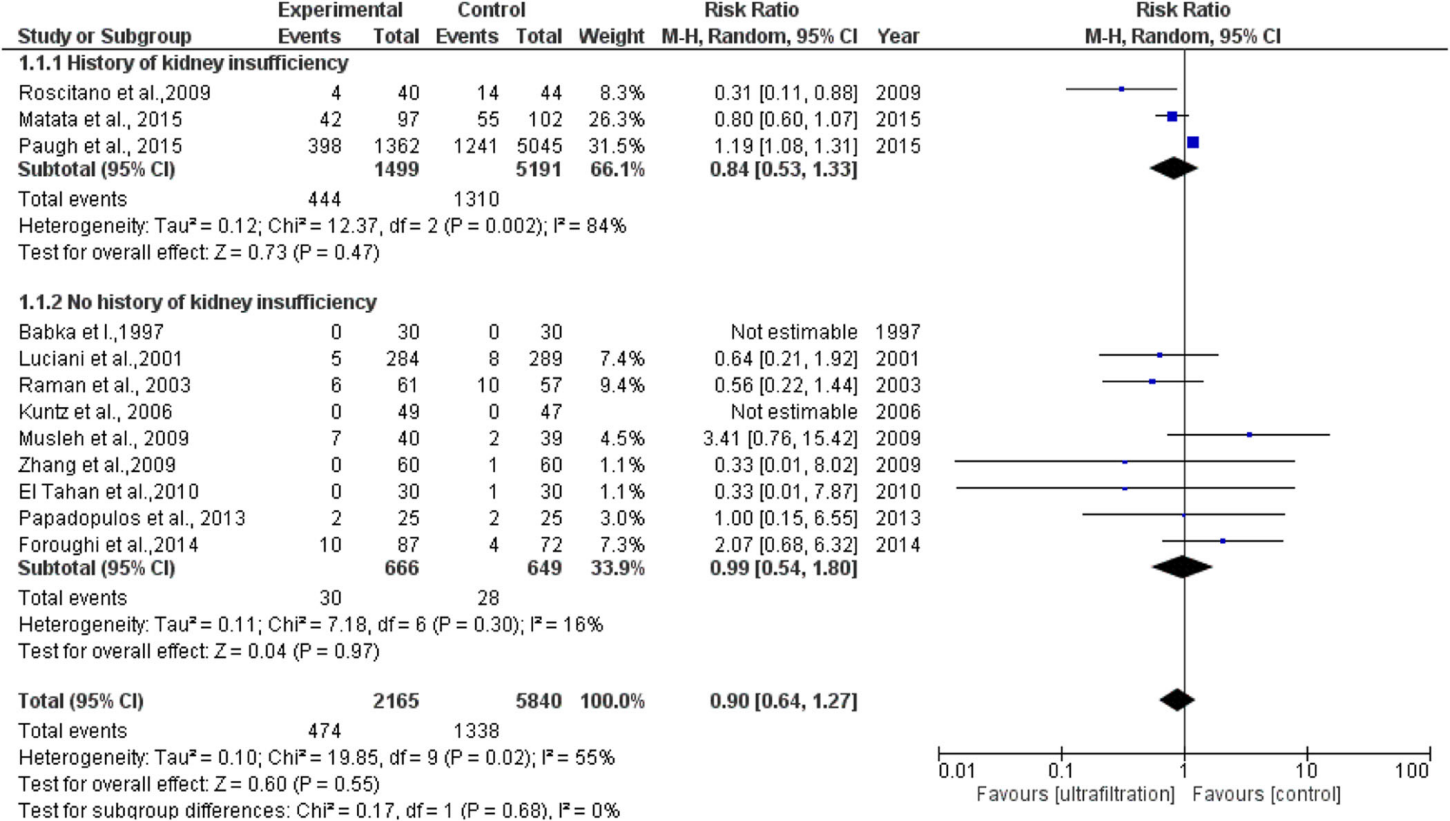
UF and AKI subgroup analysis according to the history of kidney insufficiency or not. AKI, acute kidney injury; UF, ultrafiltration

The total number of patients included in the meta‐analysis in the ultrafiltration group is 2165 patients (mean age: 62.8) and the total number of patients in the control group is 5840 patients (mean age: 61.7). The total number of patients who developed AKI in the ultrafiltration group is 1779 and the total number of patients who developed AKI in the control group is 1338. The pooled analysis between both groups was (RR = 0.90, 95% CI = 0.64–1.27, *p* = .55). We observed no publication bias among the included studies, as shown in a figure in the supplemental information. We observed heterogeneity among the included studies that were not solved by random effects (*p* = .02), as shown in Figure 2A, so we did omit one study^15^ from analysis and the heterogeneity solved and *p* value of heterogeneity became .21 and the pooled analysis became (RR = 0.79, 95% CI = 0.52–1.20 *p* = .28) as shown in Figure 2B. We performed subgroup analysis based on three factors (type of technique used, quantity of volume removed, and history of kidney insufficiency). Type of the technique used included: MUF, CUF, combined CUF and MUF, and ZBUF subgroups. The pooled analyses between the ultrafiltration group and the control group in MUF, CUF, combined MUF and CUF and showed no significant difference in AKI incidence, as shown in Figure 3A. Quantity of volume removed was divided into two subgroups (less than 2900 ml and more than 2900 ml). The pooled analyses between ultrafiltration group and control group in volume <2900 ml and volume >2900 ml subgroups were (RR = 1.12, 95% CI = 0.78–1.61, *p* = .54) and (RR = 0.82, 95% CI = 0.63–1.07, *p* = .15), respectively, as shown in Figure 3B. The history of kidney insufficiency was divided into two subgroups (history of kidney insufficiency and no history of kidney insufficiency). The pooled analyses between the UF group and control group in history of kidney insufficiency and no history of kidney insufficiency subgroups were (RR = 0.84, 95% CI = 0.53–1.33, *p* = .47) and (RR = 0.99, 95% CI = 0.54–1.80, *p *= .97), respectively, as shown in Figure 4. After doing subgroup analysis, we observed no heterogeneity in each subgroup except in two subgroups only (CUF and history of kidney insufficiency) out of the eight subgroups.

## 4 DISCUSSION

Our analysis found no significant difference in AKI incidence between patients undergoing cardiac surgery having undergone UF and those who have not (RR = 0.90, 95% CI = 0.64–1.27, *p* = .55). We also found no increase in AKI incidence in studies that removed an ultrafiltrate volume above what was set to be a critical value of 2900 ml (RR = 0.82, 95% CI = 0.63–1.07, *p*  = .15).

We did also subgroup analysis according to the type of ultrafiltration procedure performed and again no significant difference in AKI incidence between ultrafiltration groups and controls was observed in either the MUF, CUF, ZBUF, and combined CUF and MUF.

Our results (RR = 0.84, 95% CI = 0.53–1.33, *p* = .47) contradict some studies that claim the UF increase AKI risk in patients with previous kidney disease like Paugh^15^ in which the rate of AKI was higher in the filtration group and another study Musleh^13^ where there is observed a higher number of AKI in the filter group. It is important to mention that even in these two studies although there is an observed increase in AKI incidence, patients have similar rates of death, postoperative length of stay, and readmissions compared to the group who did not undergo ultrafiltration, so this claimed dysfunction does not really affect the clinical outcome and patients did not require either dialysis or support to treat their AKI.

Moreover, a study conducted in Turkey demonstrated no difference between two groups with and without filtration regarding postoperative serum creatinine, which agrees with our findings. Furthermore, serum creatinine even decreased in the filter group.^18^

In patients with kidney insufficiency, special precautions need to be taken in the preoperative period to correct some problems that may affect the surgery's outcome and increase AKI's risk independent of the UF, such as hyperkalemia. Thus, a possible explanation for the increase in AKI observed in some studies cited here may be due to the neglect of these precautions.

An important point to consider is the amount and type of CPB circuit priming solution used. Regarding the type, isotonic saline can cause renal vasoconstriction and worsened renal function meanwhile balanced crystalloid solutions are better choices due to their abilities to achieve physiologic electrolyte concentrations and reduce renal complications.

Concerning the priming solution amount, a reduction in its volume may be translated into fewer transfusions where more homologous transfusions raise the risk of AKI.^5^

### 4.1 Implication for future practice

A feasible and easy way could be used for early detection of the slightest kidney injury using urinary biomarkers, such as IGFBP7 and TIMP2, involved in G1 cell cycle arrest, urinary PO2, or NGAL.^19^, ^20^ Also, the use of preoperative plasma GDF‐15 independently predicts postoperative AKI in patients undergoing elective cardiac surgery and is particularly helpful for risk stratification in patients even with normal creatinine.^21^

Also, cystatin C, a biomarker commonly used in practice could also predict postsurgery AKI (https://www.ahajournals.org/doi/abs/10.1161/circ.136.suppl_1.21142; https://app.covidence.org/reviews/161933).^2^, ^21^, ^22^, ^23^, ^24^, ^25^

### 4.2 Limitations of our study

Several studies we included in our analysis had not mentioned the weight indexed volume of filtrate removed so we had to run the analysis based on the total volume of filtrate removed instead.

## 5 CONCLUSION

UF in cardiac surgery is safe and does not increase the risk of AKI, even in patients with previous kidney problems. Also, the removal of a volume of filtrate above 2900 ml during the procedure was not shown to negatively affect outcomes.

## Supporting information

Supporting information.
