# Supplementary figures and images for: Ultrafiltration and cardiopulmonary bypass associated acute kidney injury: A systematic review and meta‐analysis
Source: Clin Cardiol. 2021 Nov 27;44(12):1700–8. doi: 10.1002/clc.23750 (PMC8715396; doi:10.1002/clc.23750)

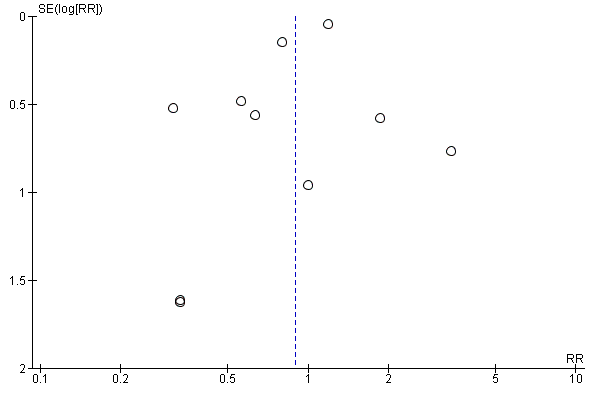


Supplementary Table

Supplement: Supplementary file 1 — Supporting information. [file CLC-44-1700-s001.docx]
